# Low-Dose Atropine for Pediatric Myopia: First-Year Progression as a Risk-Stratification and Management Signal

**DOI:** 10.1016/j.xops.2026.101300

**Published:** 2026-06-24

**Authors:** Soh Youn Suh, Karenna Mehta, Robert A. Clark

**Affiliations:** 1 Stein Eye Institute, University of California–Los Angeles, Los Angeles, California; 2 Department of Ophthalmology, David Geffen School of Medicine at UCLA, Los Angeles, California; 3 South Bay Family Eye, Long Beach, California

**Keywords:** Myopia control, Atropine, Adherence, Treatment escalation

## Abstract

**Purpose:**

To determine whether first-year refractive progression predicts longer-term on-treatment myopia control and whether early progression guides escalation decisions and subsequent trajectories.

**Design:**

Retrospective cohort, single practice.

**Participants:**

Five hundred fifty-five children (1107 eyes) treated with 0.01% (93.5%) or 0.02% (6.5%) atropine for ≥12 months.

**Methods:**

Spherical equivalent refraction (SER) progression was annualized (diopters [D]/year; positive = worse). At 12 months, eyes met or did not meet the ≤0.25 D/year threshold; children were categorized as responsive (both eyes), partially responsive (one eye), or unresponsive (neither eye). Progression was also modeled continuously (per 0.25 D/year). Escalation was defined as increasing the atropine concentration and/or adding optical therapy. Primary analyses associated 12-month response with on-treatment outcomes and progression before and after escalation. Eye-level models accounted for intereye correlation and baseline covariates. One-eye-per-subject sensitivity analyses were also performed.

**Main Outcome Measures:**

Successful control (≤0.25 D/year over on-treatment course) and change in SER progression around escalation.

**Results:**

Baseline age was 9.8 ± 2.8 years and baseline SER –2.34 ± 2.37 D. 340 (61.3%) children were responsive, 99 (17.8%) partially responsive, and 116 (20.9%) unresponsive, with successful control rates of 70%, 28%, and 17%, respectively. 12-month status strongly predicted whole-course successful control in bilateral generalized estimating equations (odds ratio [OR]: 5.71; 95% confidence interval [CI]: 3.85–8.47) and one-eye sensitivity (OR: 10.90; 95% CI: 6.32–18.80) analyses and slower whole-course progression (β –0.27 D/year, 95% CI: –0.31 to –0.23). Each +0.25 D/year of first-year progression reduced success odds (OR: 0.55; 95% CI: 0.48–0.63). Post-12-month predictive value was attenuated relative to the whole-course analyses in both the bilateral generalized estimating equations (OR: 1.32; 95% CI: 0.94–1.86; *P* = 0.110) and one-eye sensitivity (OR: 1.92; 95% CI: 1.23–3.01; *P* = 0.004) analyses. Escalation occurred in 270 (48.6%) children, earlier among unresponsive children (log-rank *P* < 0.001). Event-time analyses showed marked slowing after first atropine escalation in initial failure eyes (0.71 → 0.28 D/year; Δ –0.43; *P* < 0.001) but minimal change in initial success eyes (0.189 → 0.193 D/year; Δ +0.004; *P* = 0.892). With atropine monotherapy, early response modestly predicted post-12-month success (OR: 1.57; 95% CI: 1.02–2.41).

**Conclusions:**

Twelve-month response strongly stratifies overall on-treatment control as an early management signal, not a fixed treatment end point. Response-guided escalation slowed initial failures and attenuated post-12-month differences.

**Financial Disclosure(s):**

Proprietary or commercial disclosure may be found in the Footnotes and Disclosures at the end of this article.

The rapidly increasing prevalence of childhood myopia during the past few decades represents a significant global health concern.^1^^,^^2^ Based on one analysis, the number of individuals with myopia is predicted to reach 4.76 billion, affecting nearly 50% of the world's population.^2^ The progression of myopia can result in high myopia, which increases the risk of serious, lifelong visual impairments, including myopic maculopathy, retinal detachment, and glaucoma.^3^, ^4^, ^5^, ^6^ Beyond the visual consequences, the need for ongoing corrective lenses and potential surgical interventions places a substantial economic burden on individuals, families, and health care systems.^7^, ^8^, ^9^ Slowing myopia progression during childhood is critical to mitigating these long-term health and financial consequences.

Multiple randomized controlled studies have demonstrated that myopia control interventions can slow progression, including low-dose atropine^10^, ^11^, ^12^, ^13^, ^14^, ^15^ and specific optical modalities such as multifocal contact lenses^16^^,^^17^ and peripheral-defocus lenses.^18^, ^19^, ^20^ Clinical myopia management is dynamic. When atropine is part of the treatment plan, clinicians must decide not only whether and when to initiate it but also when to escalate, when to de-escalate or discontinue, and how to monitor for possible progression acceleration after tapering. We define escalation here as either increasing the atropine concentration or incorporating an optical myopia-control modality into the management plan. Treatment sequencing varies across practices, and these longitudinal management decisions are rarely captured in a single fixed-regimen trial framework, which estimates efficacy under predetermined treatment assignment but does not reflect how clinicians might modify therapy once first-year progression becomes known.

A central challenge in routine care is variable on-treatment progression. Even when population-level efficacy is established, individual children vary widely in progression rates during treatment, reflecting in part differences in underlying baseline progression risk that predate any intervention. Contributing factors include differences in atropine formulation and bioavailability, variability in adherence to the prescribed dosing regimen, rebound dynamics following treatment cessation, and heterogeneity in cohort composition and management structure across practice settings. In practice, early progression is often used as a management signal to guide counseling and escalation: children with faster progression are preferentially shifted to perceived higher efficacy modalities, while children with good control usually remain on the initial treatment regimen. This response-guided escalation can attenuate or “compress” later outcome differences between initially strong and initially poor responses, complicating interpretation of long-term whole-course effectiveness if early response status is treated as an immutable label. In many practices, refractive outcomes—most commonly spherical equivalent refraction (SER)—remain the most consistently available metric guiding these decisions, even as axial length measurement is increasingly emphasized in controlled studies. Recent evidence demonstrates imperfect coupling between SER and axial length across pediatric population-based studies, supporting SER as an independent and clinically meaningful metric rather than merely a surrogate for axial length.^21^

Whether 12-month refractive response should be interpreted as a fixed treatment success/failure end point or as an actionable management signal that identifies children who may benefit from a change to higher efficacy modalities remains unclear. To better define how established efficacy from clinical trials translates into real-world effectiveness under adaptive management, we analyzed a large, single-center US cohort of children treated with low-dose atropine for progressive myopia. Because the clinical meaning of 12-month refractive change lies in how it informs subsequent management, we asked 2 related questions: first, whether the initial 12-month refractive response predicts overall on-treatment outcomes and outcomes after the 12-month landmark assessment; and second, how response-guided management after that assessment—including escalation, adherence, tapering, and more uniform monotherapy management—shapes subsequent progression trajectories in routine clinical practice.

## Methods

### Study Design and Ethics

We analyzed a retrospective cohort of children treated for progressive myopia with low-dose atropine at a single US ophthalmology practice, using data collected between 2012 and 2025. Providence Institutional Review Board approval was obtained, and a waiver of consent was granted for this retrospective chart review. All research procedures adhered to the tenets of the Declaration of Helsinki and applicable privacy regulations.

### Participants

**Figure 1:**
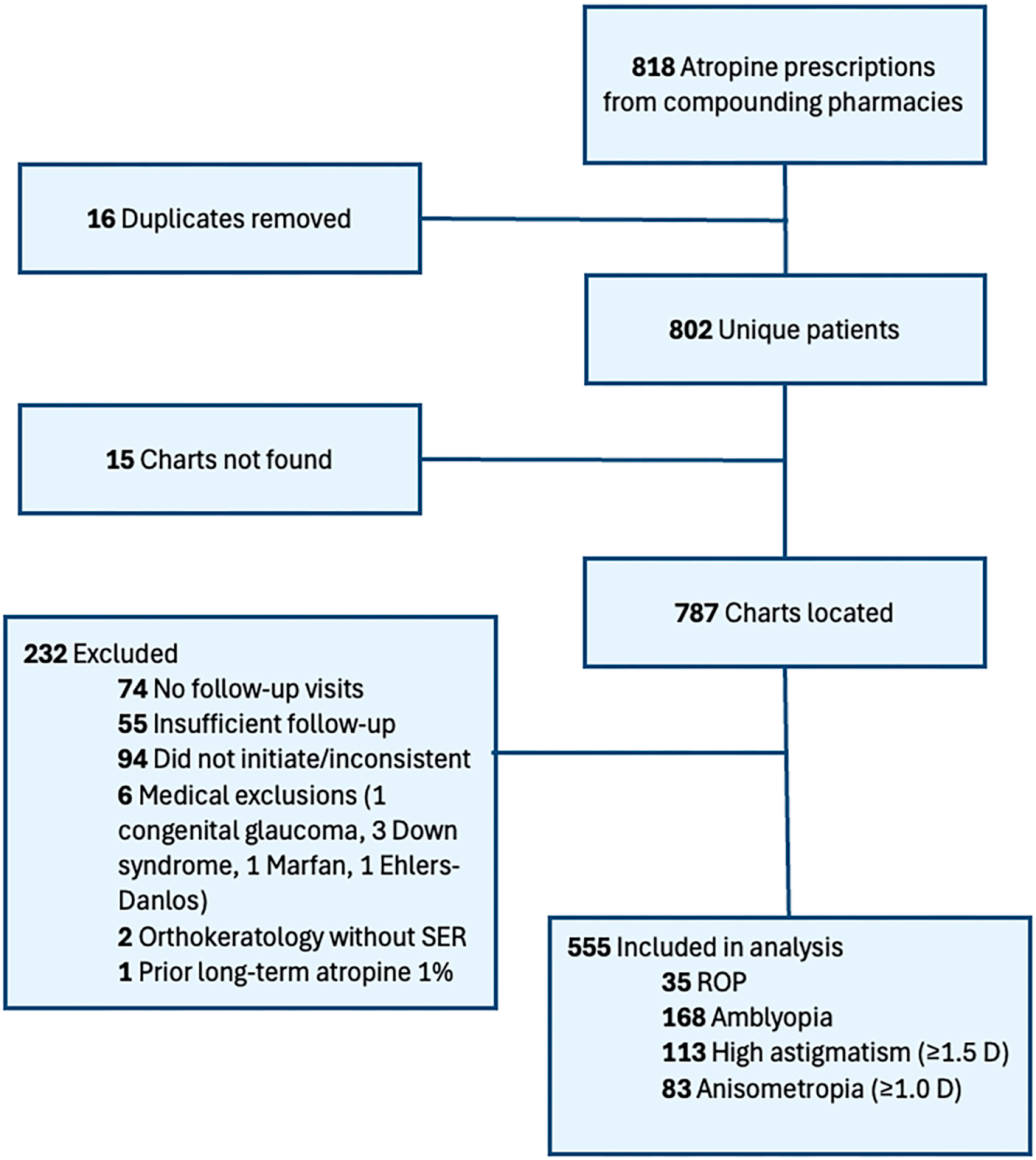
Flow diagram of participants. D = diopters; ROP = retinopathy of prematurity; SER = spherical equivalent refraction.

We reviewed 787 charts of children prescribed low-dose atropine. Progressive myopia was defined pragmatically as clinically documented ongoing myopic SER based on available refraction history at the time of atropine initiation. Uniform pretreatment progression data were not available for all children and therefore were not used as a formal quantitative inclusion criterion. We excluded 232 cases: no follow-up (n = 74), insufficient follow-up (n = 55), never started or persistent inconsistent use sufficient to preclude meaningful interpretation of treatment effect in routine care (n = 94), orthokeratology with missing SER data (n = 2), prior long-term 1% atropine (n = 1), or conditions affecting refraction (congenital glaucoma, n = 1; Down syndrome, n = 3; Marfan/Ehlers-Danlos, n = 2). The final cohort was 555 children (1107 eyes; 3 children contributed 1 treated eye—2 with anisometropic hyperopia and 1 with unilateral surgical aphakia) with at least 12 ± 3 months of follow-up (Fig 1). Because self-identified race/ethnicity was not consistently available in this retrospective chart review, Bayesian Improved Surname Geocoding (BISG) was used to provide probabilistic surname-based and geocoding-based estimates for covariate adjustment. Bayesian Improved Surname Geocoding does not substitute for self-identified race/ethnicity, and individual-level misclassification is possible.

This cohort reflects a private pediatric-ophthalmology referral practice rather than an uncomplicated community myopia sample. Two referral streams explain the observed comorbidity pattern. Younger children were commonly referred after failed vision screening, with primary diagnoses of hyperopia, astigmatism, anisometropia, or amblyopia; many subsequently developed progressive myopia or presented with concurrent low-to-moderate myopia and became atropine candidates. Older children were more often referred specifically for corrected visual acuity (VA) below 20/20, a population enriched for myopia with astigmatism. This 2-stream composition explains the relatively high and frequently overlapping prevalences of high astigmatism, anisometropia, and refractive amblyopia in the cohort.

### Interventions

Initial therapy for all children consisted of either atropine 0.01% (519/555, 93.5%) or atropine 0.02% (36/555, 6.5%), prescribed at physician discretion. Optical therapies, including dual focus contact lenses (DF-CTL) (MiSight, CooperVision, Inc) and/or peripheral-defocus glasses (PD-Glasses) (MyoZoom, Riccino Optical, Inc), were used as adjunctive treatment when additional intervention was thought to be necessary, either initiated at baseline (DF-CTL: 34/555, 6.1%; PD-Glasses: 10/555, 1.8%) or added during follow-up. Escalation was defined as any increase in atropine concentration up to 0.05% and/or the addition of an optical modality and was analyzed descriptively. Event-time analyses were aligned to the first atropine concentration escalation, and separate event-time analyses examined initiation of DF-CTL or PD-Glasses after baseline. De-escalation was defined as any tapering of atropine concentration or cessation of therapy and was undertaken at physician discretion, typically in children with sustained good control for at least 1 year and/or in older adolescents with apparent myopia stabilization. Children were generally seen every 6 months, with decisions regarding continuation, escalation, tapering, or cessation made at physician discretion at each visit based on observed progression; for analyses, the principal landmark assessment was the 12-month visit, and post-12-month analyses required at least 1 year of follow-up beyond that landmark.

Adherence and side effects (photophobia, irritation, and blurred vision) were recorded at every visit. Atropine was prescribed as once-nightly application to each eye undergoing treatment. Adherence was family-reported (1 = consistent use, 0 = inconsistent use), averaged across visits, with >0.9 defined as good adherence, ≥0.7 to ≤0.9 as moderate adherence, and <0.7 as poor adherence. Side effects were coded as present or absent.

### Outcome Measures

Spherical equivalent refraction was defined as sphere + ½ cylinder, using the refraction method documented during routine care (retinoscopy with or without cycloplegia or manifest refraction in a subset of older children, with consistent techniques maintained for each child). Progression rates were recorded as positive for worsening myopia (progression rate = [SER (start) – SER (end)]/years).

Early response was assessed at the 12-month visit (12 ± 3 months after treatment initiation): eyes were classified as initial success if progression was ≤0.25 diopters (D); otherwise, eyes were classified as initial failures. This threshold corresponds to one standard clinical refraction increment and was used as a practical clinical-action boundary rather than a biologically validated breakpoint.^22^ Children were classified as responsive (both eyes initial successes), partially responsive (one eye initial success), or unresponsive (both eyes initial failures). Throughout the manuscript, the term “response” refers to this 12-month annualized classification (≤0.25 D/year vs. >0.25 D/year) and does not denote maintenance of that threshold over the whole on-treatment course.

The primary outcome was eye-level successful control, defined as overall SER progression ≤0.25D/year from treatment initiation to the visit when atropine was stopped or the last available follow-up visit if no discontinuation was recorded, restricted to children with ≥2 years of on-treatment follow-up. Bilateral (subject-level) control required successful control in both eyes. Secondary outcomes examined progression from the 12-month visit onward when the post-12-month follow-up was ≥1 year, with success again defined as ≤0.25 D/year.

### Statistical Analysis

Analyses were performed at the eye level with adjustment for intereye correlation using R (v4.5.1).^23^ We used generalized estimating equations (GEE) with exchangeable correlation structure: Gaussian for continuous progression rates and binomial for binary success outcomes. Models adjusted for baseline age, baseline SER, baseline atropine concentration, sex, predicted ethnicity, and comorbidity indicators (high cylinder, defined as any documented cylinder ≥1.5 D from baseline through follow-up, and retinopathy of prematurity [ROP] severity as exploratory covariates). We also performed a one-eye-per-subject sensitivity analysis (right eye preferred; left eye only if unilateral). To address concerns about dichotomization, we repeated key analyses modeling first-year progression continuously (per +0.25 D/year) as a complementary analysis of the graded biologic association and performed additional sensitivity analyses for atropine-monotherapy eyes without escalation.

To assess whether the main signal was confined to a more trial-eligible subgroup, we performed an exploratory initial 12-month status × Pure_Group interaction analysis for whole-course successful control, whole-course progression, and post-12-month success. Pure_Group was defined by strict trial-style criteria: baseline age 6 to 18 years, baseline SER –0.50 to –8.00 D, no amblyopia, no high astigmatism, no anisometropia, no ROP, and no optical exposure. Because several defining variables were components of group membership, these models used a reduced covariate set (baseline age, baseline SER, sex, and predicted ethnicity). A parallel no-optical-ever interaction sensitivity analysis was also performed.

Time-to-first escalation was evaluated using Kaplan-Meier methods and Cox proportional hazards models. To test whether postescalation slowing reflected treatment-associated change rather than regression toward the mean (RTM), we used event-time analyses aligned to the first atropine concentration escalation (k = 0), cylinder change as a negative-control outcome, and a placebo/lead test (unusually fast progression predicting future escalation). Similar event-time analyses were performed around postbaseline initiation of DF-CTL or PD-Glasses.

## Results

### Baseline Characteristics

A total of 555 children (1107 eyes) were included with a mean follow-up period of 3.5 ± 2.4 years (range: 0.8–12.4 years). Baseline characteristics included mean age of 9.8 ± 2.8 years (range: 0.4–18.8 years), mean SER of –2.34 ± 2.37 D (range: +0.63 to –18.13 D), median age was 9.7 years (interquartile range [IQR]: 7.7–11.8), and median SER was –1.75 D (IQR: –0.75 to –3.25 D). Females comprised 51.2% of the cohort. Ethnicity was predicted as 21.3% Asian, 19.5% Hispanic, and 59.3% Caucasian/mixed. Most children (93.5%) were initially prescribed 0.01% atropine, while 6.5% received 0.02%. Adjunctive optical therapies included DF-CTL (n = 109 [19.6%]; 34 [6.1%] started at baseline) and peripheral-defocus spectacles (n = 71 [12.8%]; 10 [1.8%] started at baseline). For comorbidities, amblyopia was present in 168 children (30.3%; refractive = 147, strabismic = 21), high astigmatism in 113 (20.4%; 89 at baseline), and anisometropia in 83 (15.0%). Additionally, 35 children (6.3%) had a history of ROP (stage 0 = 21, stage 2 = 3, stage 3 = 11) (10 laser, 2 anti-VEGF [1 child received both treatments]) (Table 1).

**Table 1:** Cohort Characteristics by Initial 12-Mo Response Category

Characteristic (Subjects)	Overall (555)	Responsive (340, 61.3%)	Partially Responsive (99, 17.8%)	Unresponsive (116, 20.9%)
Baseline age, yrs	9.8 ± 2.8	10.2 ± 2.8	9.0 ± 2.6	9.3 ± 2.5
Females	284 (51.2%)	175 (61.6%)	46 (16.2%)	63 (22.2%)
Predicted Asian	118 (21.3%)	70 (59.3%)	24 (20.3%)	24 (20.3%)
Predicted Hispanic	108 (19.5%)	65 (60.2%)	22 (20.4%)	21 (19.4%)
Predicted Caucasian/mixed	329 (59.3%)	205 (62.3%)	53 (16.1%)	71 (21.6%)
Baseline SER (eye), diopters	–2.34 ± 2.37	–2.31 ± 2.50	–2.34 ± 2.38	–2.44 ± 1.91
Baseline atropine 0.01%	519 (93.5%)	318 (61.3%)	91 (17.5%)	110 (21.2%)
Baseline atropine 0.02%	36 (6.5%)	22 (61.2%)	8 (22.2%)	6 (16.7%)
DF-CTL at baseline	34 (6.1%)	23 (67.6%)	6 (17.6%)	5 (14.7%)
PD-Glasses at baseline	10 (1.8%)	4 (40.0%)	2 (20.0%)	4 (40.0%)
DF-CTL any time	109 (19.6%)	62 (56.9%)	19 (17.4%)	28 (25.7%)
PD-Glasses any time	71 (12.8%)	40 (56.3%)	13 (18.3%)	18 (25.4%)
Amblyopia	168 (30.3%)	93 (55.4%)	33 (19.6%)	42 (25.0%)
High astigmatism	113 (20.4%)	60 (53.1%)	22 (19.5%)	31 (27.4%)
Anisometropia	83 (15.0%)	35 (42.2%)	24 (28.9%)	24 (28.9%)
Retinopathy of prematurity	35 (6.3%)	19 (54.3%)	5 (14.3%)	11 (31.4%)
Years on atropine	3.5 ± 2.4	3.6 ± 2.3	3.4 ± 2.4	3.2 ± 2.4

DF-CTL = MiSight contact lens; PD-Glasses = MyoZoom spectacle lens; SER = spherical equivalent refraction.

Overall column values are n (% of cohort). Values in the response-category columns are n (row %), representing the distribution of response status within that characteristic (e.g., among Asian children, 59.3% were responsive). Header percentages show the proportion of the full cohort in each response category. Baseline SER is summarized at the eye level (1107 eyes); other characteristics are summarized at the subject level (555 children). Predicted ethnicity was estimated via Bayesian Improved Surname Geocoding (BISG). High astigmatism was any documented cylinder ≥1.5 diopters from baseline through follow-up. Anisometropia was ≥1.0 diopters difference between eyes at baseline.

### Early Response and Overall Treatment Success

At the 12-month visit, 61.3% (n = 340) were responsive, 17.8% (n = 99) partially responsive, and 20.9% (n = 116) unresponsive (Table 1). For the primary success analysis, 402/555 (72.4%) children met the follow-up requirement with ≥2 years on treatment. Compared with the full cohort, these 402 children had longer follow-up (mean: 4.76 ± 2.35 years; median: 3.99, IQR: 3.02–6.09) and slightly younger mean baseline age (9.55 ± 2.70 vs. 9.80 ± 2.79 years). For those children, mean SER progression and rates of successful control differed strongly by early response category (χ^2^ test, *P* < 0.0001, Table 2). Initial 12-month status strongly predicted whole-course success: positive predictive value = 0.70 (95% confidence interval [CI]: 0.64–0.75) for initial successes and negative predictive value = 0.77 (95% CI: 0.70–0.83) for initial failures. These predictive values apply to the overall on-treatment course, which included the first year and subsequent response-guided management, and therefore differ from the post-12-month visit analyses below, which isolate outcomes after the 12-month landmark assessment. Baseline SER (*P* = 0.87) and follow-up duration (*P* = 0.22) did not differ significantly between groups.

**Table 2:** Successful Control by Initial 12-Mo Status (≥2 Yrs on Treatment)

Response Category	Subjects	Successful Control	Mean Annualized Progression, D/yr
Responsive	243	171 (70.4%)	0.11 ± 0.21
Partially responsive	78	22 (28.2%)	0.28 ± 0.21
Unresponsive	81	14 (17.3%)	0.44 ± 0.28

Of 555 subjects, 402 had ≥2 yrs on treatment. Initial 12-mo status was defined by spherical equivalent refraction (SER) progression at 12 mo: SER ≤0.25 diopters (D)/yr defined eye-level initial success and >0.25 D/yr defined eye-level initial failure, while subject-level response category was defined as responsive (both eyes initial success), partially responsive (one eye initial success), and unresponsive (both eyes initial failure). Success was defined as ≤0.25 D/yr in all observed eyes for the duration of treatment. Mean annualized progression is summarized at the eye level.

In multivariable bilateral eye-level GEE models, initial 12-month status strongly predicted success over the entire treatment course (odds ratio [OR]: 5.71; 95% CI: 3.85–8.47; *P* < 0.001); initially successful eyes progressed 0.27 D/year less on average (β = –0.27; 95% CI: –0.31 to –0.23). Continuous modeling for initial 12-month progression confirmed a gradient effect: each 0.25 D/year worse progression reduced the odds of whole-course successful control (OR: 0.55; 95% CI: 0.48–0.63). In the one-eye-per-subject sensitivity analysis, initial response remained strongly associated with whole-course successful control (OR: 10.90; 95% CI: 6.32–18.80; *P* < 0.001). Older baseline age increased odds of success (OR: 1.15 per year; *P* = 0.001), while baseline SER, sex, and predicted ethnicity were not significant predictors. At baseline, initial-success eyes were on average older than initial-failure eyes (10.05 ± 2.80 vs. 9.20 ± 2.66 years; *P* < 0.001). Sensitivity to visit gaps showed no differential flagging by responder status (both ∼1%).

### Post-12-Month Visit Outcomes

Because treatment was frequently escalated after the 12-month visit, particularly in initial failures, we evaluated whether early response predicted outcomes in the post-12-month visit window. The post-12-month analytic subset comprised 447 children with ≥1 year of follow-up after the 12-month landmark (mean post-12-month follow-up: 3.46 ± 2.34 years; median: 2.75, IQR: 1.81–4.79; mean total follow-up from baseline: 4.54 ± 2.34 years). For SER progression from the 12-month visit until treatment cessation, restricted to children with ≥1 year follow-up after the 12-month visit, early response did not predict progression (β 0.00 D/year; 95% CI: –0.05 to 0.05; *P* = 0.98). For post-12-month success, predictive value was attenuated relative to the whole-course analyses in both the bilateral GEE (OR: 1.32; 95% CI: 0.94–1.86; *P* = 0.110) and one-eye sensitivity analyses (OR: 1.92; 95% CI: 1.23–3.01; *P* = 0.004). We retain the bilateral GEE model as primary for this bilateral longitudinal data set and report the one-eye analysis as a sensitivity analysis. High astigmatism was associated with lower odds of successful post-12-month visit control (OR: 0.61; 95% CI: 0.39–0.94; *P* = 0.026).

To provide a clearer clinical view of what happened after the 12-month decision point in the dominant baseline treatment group, we performed a descriptive analysis restricted to children who began on 0.01% atropine, had no baseline optical therapy, and had both 12±3-month and 24±3-month follow-up available (n = 305). Children were grouped by 12-month subject status (responsive, partially responsive, or unresponsive) and by whether treatment was escalated after 12 months (Table 3). For example, a child with no SER change from baseline to the 6-month visit but 0.25 D progression between 6 and 12 months has an annualized 12-month progression rate of 0.25 D/year (still meeting the responsive threshold) but an apparent acceleration on the second-half slope that often prompted escalation. Such children are categorized as responsive by the annualized 12-month rule but appear in the responsive-escalated cell of Table 3. In responsive children, mean progression increased from year 1 to year 2 both without escalation (0.11–0.23 D/year; Δ +0.12) and with escalation (0.04 to 0.29 D/year; Δ +0.25). The low year-1 progression in the responsive-escalated group (0.04 D/year) reflects this within-year trajectory pattern. In partially responsive children, mean progression decreased from year 1 to year 2 both without escalation (0.30 to 0.23 D/year; Δ –0.07) and with escalation (0.40–0.22 D/year; Δ –0.18). In unresponsive children, year-2 progression also decreased substantially, with a larger descriptive reduction in the escalated group (0.90 to 0.32 D/year; Δ –0.58) than in the nonescalated group (0.75–0.36 D/year; Δ –0.39). Although RTM cannot be excluded from a descriptive table, pure RTM would predict similar year-2 trajectories in escalated and nonescalated children within each status category; the consistent within-category differential favoring the escalated group, most pronounced in unresponsive children (0.19 D/year greater reduction), is directionally compatible with a treatment contribution beyond expected mean reversion. Because escalation was selectively used in children with greater clinical concern, this table is nonetheless presented descriptively rather than as a causal comparison of escalation strategies.

**Table 3:** Change in Myopia Progression From Year 1 to Year 2 by 12-Mo Status and Post-12-Mo Escalation Baseline 0.01% Atropine, Baseline-Optical-Free Cohort (n = 305)

12-Month Status	Escalation after 12 Mo	Subjects	Mean Year-1 Progression, D/yr	Mean Year-2 Progression, D/yr	Change in Progression, Year 2 – Year 1, D/yr
Responsive	No escalation	172	0.11	0.23	+0.12
Responsive	Any escalation	16	0.04	0.29	+0.25
Partially responsive	No escalation	39	0.30	0.23	–0.07
Partially responsive	Any escalation	12	0.40	0.22	–0.18
Unresponsive	No escalation	41	0.75	0.36	–0.39
Unresponsive	Any escalation	25	0.90	0.32	–0.58

D = diopter.

Positive values indicate worsening myopia. Year-1 progression and Year-2 progression are both expressed using the manuscript sign convention (positive = worse). “Any escalation” includes atropine-only escalation and atropine escalation plus added optical therapy. This table is descriptive; escalation was selectively used in children with greater clinical concern.

### Management Patterns and Escalation

Treatment escalation (higher atropine concentration and/or added optical therapy) occurred in 48.6% (270/555) of children and was initiated earlier in unresponsive than in responsive children (median 2.16 vs. 4.95 years; log-rank *P* < 0.001). Escalation was more common in unresponsive (76/116, 65.5%) than in responsive children (137/340, 40.3%). The most frequent first escalation was increased atropine concentration (199/555, 35.9%).

**Figure 2:**
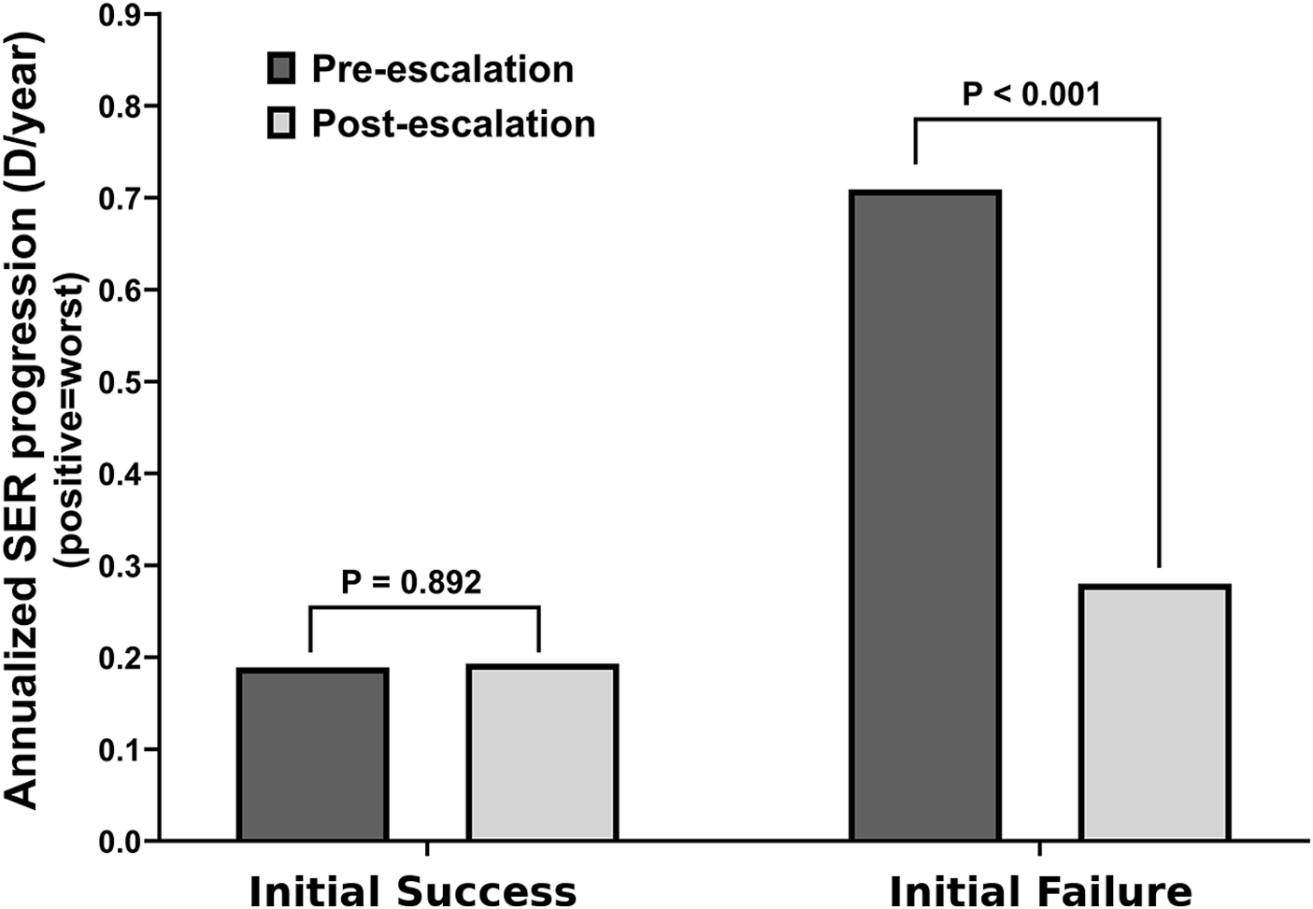
Annualized spherical equivalent refraction (SER) progression (diopters [D]/year; positive values indicate worsening myopia) before vs. after atropine escalation, stratified by initial 12-month status. Bars show mean annualized SER progression in time frames immediately pre-escalation and postescalation around the first documented atropine concentration escalation. Escalation had minimal effect on myopia progression in initial success eyes (0.189 D/year → 0.193 D/year), but dramatically slowed myopia progression in initial failure eyes (0.71 D/year → 0.28 D/year).

Event-time analyses aligned to first atropine concentration escalation showed substantial slowing among initial failure eyes (0.71 → 0.28 D/year; Δ –0.43; *P* < 0.001) but minimal change in initial success eyes (0.189 → 0.193 D/year; Δ +0.004; *P* = 0.892) (Fig 2). Difference-in-differences models confirmed significantly greater postescalation slowing in initial failures (0.39–0.43 D/year reduction; interaction *P* < 0.001). Supporting analyses reduced the possibility of RTM as the cause (placebo/lead test *P* = 0.521) and confirmed myopia-specific change (no parallel step-change in cylinder/astigmatism).

Similar patterns emerged after optical escalation. Postbaseline DF-CTL initiation produced marked slowing in 52 initial failure eyes (Δ ≈ –0.41 D/year when added to 32 eyes without concurrent atropine increase; *P* < 0.010), with little change in 67 initial success eyes (55 without concurrent atropine increase, *P* = 0.34); benefit magnitude differed significantly by initial 12-month response (difference-in-differences *P* < 0.001). PD-Glasses findings were directionally consistent but limited by smaller numbers (22 eyes total: 10 initial failures, 12 initial successes). Responsive children started DF-CTL at older ages (median 13.8 vs. 11.3 years for unresponsive children), consistent with elective rather than escalation-driven use in well-controlled cases.

### Adherence and Side Effects

Adherence significantly interacted with initial 12-month status. In initial success eyes, moderate and good adherence were associated with modestly slower progression (0.22–0.28 D/year less; *P* < 0.001), whereas in initial failure eyes, better (moderate/good) adherence correlated with faster progression (0.13–0.16 D/year more; *P* < 0.001; interaction *P* < 0.01), suggesting a pattern consistent with intrinsic nonresponse in a subset of children rather than inadequate adherence as the primary explanation for poor treatment response.

Side effects were reported by 41/555 children (7.4%), with 20/555 (3.6%) discontinuing atropine secondary to side effects; photophobia was the most common reason for discontinuation (10/20), followed by burning/irritation (8/20) and blurred vision (2/20). At the eye level, side effects were associated with slightly faster progression (β = +0.07 D/year; *P* = 0.043).

### De-Escalation and Rebound Risk

A total of 118/555 (21.3%) children underwent treatment de-escalation. Among these children, mean age at de-escalation was 13.1 ± 2.9 years and mean SER at the de-escalation visit was –3.41 ± 2.42 D, consistent with an older, more clinically stable subset of the broader cohort. Rebound was defined operationally as acceleration of myopic progression after cessation relative to the immediate precessation interval rather than merely any postcessation myopic shift. The primary rebound test was a cessation-adjacent bilateral eye-level GEE comparing the immediate precessation interval with the first off-atropine interval; the off-period coefficient was +0.04 D/year (standard error 0.04; *P* = 0.222), indicating no statistically significant acceleration immediately after cessation. No clinical predictors of rebound were identified.

### Impact of Comorbidities

High astigmatism increased myopia progression (β = +0.09 D/year; *P* = 0.002) and reduced the odds of meeting the initial success threshold (OR: 0.65; *P* = 0.013). Eyes with high cylinder had mildly reduced baseline corrected VA compared with eyes without high cylinder (mean LogMAR 0.133 [approximately 20/27 Snellen] vs. 0.052 [approximately 20/23 Snellen]), consistent with the astigmatic amblyopia expected in this group. While axial length data were not available to partition corneal from axial contributions to progression in this subgroup, the modest VA difference contextualizes the refractive findings. In exploratory analyses, severe ROP (stage 3) showed signals of better control and slower progression (*P* = 0.006). Amblyopia had minimal association with progression (*P* = 0.85). Because this referral-practice cohort included many children who would typically be excluded from randomized myopia-control trials, we also performed exploratory reason-specific subgroup analyses. The whole-course progression signal associated with initial 12-month status remained statistically significant and directionally consistent in amblyopia (β –0.31, 95% CI: –0.38 to –0.23; *P* < 0.001), anisometropia (β –0.33; *P* < 0.001), high astigmatism (β –0.25; *P* < 0.001), and ROP (β –0.35; *P* < 0.001). For the ROP subgroup, the whole-course successful-control logistic model showed separation, so only the progression model is reported. These subgroup findings are exploratory.

To evaluate whether the primary association differed materially in a stricter trial-like subgroup, we compared pure and children with comorbidities as defined above. The initial 12-month status × Pure_Group interaction was nonsignificant for whole-course successful control (interaction OR: 0.65, 95% CI: 0.27–1.58; *P* = 0.345), whole-course progression (interaction β –0.03 D/year, 95% CI: –0.12 to 0.07; *P* = 0.585), and post-12-month success (interaction OR: 1.38, 95% CI: 0.65–2.95; *P* = 0.401). In the no-optical-ever interaction sensitivity, all 3 interaction terms remained nonsignificant (*P* = 0.769, 0.833, and 0.591, respectively), supporting broad similarity rather than evidence of a fundamentally different treatment-signal population.

### Sensitivity Analyses

In a no-optical-ever sensitivity analysis restricting subjects to no dual focus contact lens or peripheral-defocus spectacle exposure at any visit, initial 12-month status remained strongly associated with whole-course successful control in the bilateral eye-level GEE model (OR: 4.47; 95% CI: 2.72–7.33; *P* < 0.001). In atropine-only subsets without treatment escalation, 12-month status modestly predicted post-12-month visit success (OR: 1.57; 95% CI: 1.02–2.41).

## Discussion

In this large, single-center US real-world cohort of 555 children treated with low-dose atropine for progressive myopia, the 12-month refractive response emerged as a useful clinical management signal rather than a fixed end point. Three findings support this practical interpretation: (1) initial 12-month status strongly stratified whole-course on-treatment control, confirming that first-year SER progression contains meaningful prognostic information; (2) when outcomes were restricted to the post-12-month window, initial response no longer predicted subsequent progression or success in the full cohort, consistent with adaptive clinical management selectively escalating faster progressors to compress longer-term differences; and (3) escalation strategies were associated with clinically meaningful slowing of progression for initial failures, with little evidence of additional benefit for initial successes. Together, these findings reinforce a key message: initial failure should not be accepted as inevitable failure to control myopia progression, because treatment escalation is associated with a meaningful reduction in progression rates in previously unresponsive children.

This cohort should be interpreted as a private pediatric-ophthalmology referral-practice data set rather than a typical uncomplicated community myopia population. Two referral streams likely underlie the observed heterogeneity: younger children referred after failed vision screening, often with hyperopia, astigmatism, anisometropia, or amblyopia before or alongside later myopic progression, and older children referred for reduced corrected VA who were enriched for more straightforward myopia. Accordingly, subgroup findings involving amblyopia, ROP, and high astigmatism should be interpreted as descriptive of this real-world population rather than as generalizable to typical uncomplicated myopia, and the manuscript's core claims are scoped to this referral-practice context. Baseline dual-therapy initiation occurred in a small subset of children at physician discretion based on clinical assessment at the initial visit; the no-optical-ever sensitivity analysis confirms the primary whole-course signal is not driven by this subset.

Initial 12-month status was highly informative when outcomes were evaluated across the entire on-treatment course. In adjusted bilateral eye-level models, initial success was associated with substantially higher odds of successful control and slower mean progression, and the whole-course association remained strong in the one-eye sensitivity analysis. These results support first-year SER progression as a pragmatic, readily available metric in routine care that can stratify overall risk and expected management burden. The 0.25 D/year threshold should therefore be interpreted as a practical clinical-action boundary rather than a precise biologic breakpoint^22^: one standard clinical refraction increment is useful for escalation decisions in routine retinoscopic care, whereas the continuous model, in which each +0.25 D/year worse first-year progression reduced whole-course success odds (OR: 0.55; 95% CI: 0.48–0.63), more directly captures the graded biologic relationship between first-year progression and long-term control. Published estimates of untreated school-age myopic progression in age-matched and ethnicity-matched populations typically exceed the 0.25 D/year threshold considerably; eyes progressing somewhat above this threshold on treatment in this cohort may therefore still be progressing more slowly than expected in the absence of treatment.

Initial 12-month status, however, showed attenuated post-12-month prognostic value relative to the whole-course analyses in the primary bilateral GEE analysis. The one-eye-per-subject sensitivity analysis yielded a larger post-12-month estimate, but prognostic value was attenuated in both analytic frameworks, with attenuation most evident in the bilateral model. This pattern is biologically and statistically plausible in bilateral longitudinal data, in which intereye discordance and subject-level management can reduce later eye-level prognostic separation and thereby narrow post-12-month differences. Clinically, this is not evidence that the initial response is “meaningless,” but rather that initial response was acted upon: children who progressed faster at 12 months were more likely to receive higher efficacy therapy, and that shift in therapy narrowed subsequent outcome differences. This distinction also clarifies why predictive values can appear strong for whole-course outcomes while prediction attenuates after the 12-month landmark. Specifically, positive predictive value/negative predictive value in this manuscript describes how 12-month response predicts whole-course bilateral control (which includes the first year and subsequent response-guided management), while the post-12-month analyses deliberately isolate outcomes after the 12-month assessment. Table 3 provides a descriptive year-1 to year-2 summary in the dominant baseline 0.01%, baseline-optical-free subgroup, stratified by 12-month subject status and whether treatment was escalated after the landmark visit. This table is intended to show the clinical trajectory after the 12-month decision point, not to provide a causal estimate of escalation effect. The within-category differential between escalated and nonescalated children—most evident in the unresponsive group (Δ –0.58 vs. Δ –0.39 D/year)—is directionally compatible with the postescalation slowing observed in the primary event-time analyses, although descriptive subgroup comparisons such as Table 3 remain susceptible to residual confounding and RTM. The separate event-time escalation analyses therefore remain the more appropriate framework for evaluating what escalation itself may have contributed.

The monotherapy sensitivity analyses help triangulate this interpretation. When restricted to atropine monotherapy without escalation, initial response modestly predicted post-12-month success. More importantly for the optical-confounding concern, the no-optical-ever bilateral GEE sensitivity analysis still showed a strong association between initial 12-month status and whole-course successful control (OR: 4.47; 95% CI: 2.72–7.33; *P* < 0.001). This indicates that the core whole-course signal holds in an atropine-only management framework. This pattern is consistent with the hypothesis that, under a more uniform regimen (i.e., fewer adaptive treatment changes as might occur in a structured clinical trial), the initial 12-month status retains prognostic value for subsequent control; in contrast, in real-world clinical practice, response-guided escalation among initial failures can reduce the apparent predictive value of the initial 12-month status for later outcomes by selectively improving trajectories in those at the highest risk.

The escalation analyses reinforce this “risk stratification signal” framework. Regression toward the mean is an expected statistical phenomenon in any step-therapy design that selects children on the basis of extreme first-year values, and we do not interpret these observational event-time findings as evidence that RTM is absent. The narrower question is whether RTM alone is sufficient to explain the magnitude and directional asymmetry of the observed postescalation changes. If RTM were dominant, both extremes would be expected to move toward the population mean: initial failure eyes should slow, but initial success eyes should also accelerate. Instead, we observed strong unidirectional slowing confined to initial failure eyes, with minimal change in initial success eyes.

Several additional findings argue that the observed pattern is not explained by RTM alone. The placebo/lead test was negative (*P* = 0.521), cylinder as a negative-control outcome showed no parallel step-change, and in the no-optical-ever subset first atropine escalation produced marked slowing in initial failure eyes (0.64 → 0.33 D/year; Δ –0.31) but essentially no change in initial success eyes (0.198 → 0.195 D/year; Δ –0.003). Taken together, these findings support a therapeutic contribution beyond expected RTM while still acknowledging that some RTM is inherent to this step-therapy design.

Our optical escalation findings extend this same severity-directed interpretation. Postbaseline initiation of DF-CTL was associated with marked slowing in initial failure eyes, including initiation without a concurrent atropine concentration increase, while initial success eyes showed no statistically significant change after DF-CTL initiation alone. The observation that responsive children started DF-CTL at older ages than unresponsive children highlights a common real-world nuance: in well-controlled children, contact lens adoption may be elective rather than escalation-driven and should not automatically be interpreted as driven by ineffective myopia control. Evidence for PD-Glasses was directionally consistent but limited by small paired samples and should be considered exploratory.

Although the manuscript's primary focus was initial 12-month status and escalation, our secondary findings on adherence and tolerability provide useful clinical context. The interaction between initial 12-month status and adherence suggests that adherence was most impactful among children predisposed to respond: higher reported adherence was associated with slower progression among initial successes, while among initial failures, higher reported adherence was paradoxically associated with faster progression. While residual confounding is possible (e.g., families of rapid progressors may report higher adherence or receive more intensive counseling), this interaction is consistent with a clinically plausible subset of relative biologic heterogeneity in which adherence alone is insufficient, and escalation should be considered when progression remains rapid despite reported adherence. Side effects were uncommon, and discontinuation due to side effects was rare, supporting the overall tolerability of low-dose atropine in routine practice.

Reassuringly, tapering or cessation was not associated with a rebound increase in progression in this cohort. Instead, progression modestly slowed after discontinuation in responsive children, a finding likely influenced by the natural age-related deceleration of myopia progression in older children and adolescents. Although differences in study design, age at cessation, and follow-up duration limit direct comparisons with prior studies, these real-world findings support cautious de-escalation in well-controlled children with continued monitoring rather than indefinite continuation of treatment in all children.

Comorbidity and pure-versus-heterogeneous analyses should be interpreted as exploratory, but they help address the concern that the main signal might be confined to trial-eligible children. Strict trial-style exclusion criteria did not identify a fundamentally different treatment-signal population in this data set: the Pure_Group interaction terms were nonsignificant across the 3 primary end points, and the no-optical-ever interaction sensitivity was similarly negative. Reason-specific subgroup models showed directionally consistent whole-course progression signals across amblyopia, anisometropia, high astigmatism, and ROP. These findings support broad similarity rather than formal equivalence. For the small ROP subgroup, the whole-course successful-control logistic model showed separation, so only the progression model is reported. All subgroup findings should be interpreted cautiously given small numbers and the exploratory nature of these analyses.

This study has limitations inherent to retrospective real-world analyses. Refraction methods were determined by routine care and were not uniformly cycloplegic, introducing potential measurement variability over time. Because many refractions were obtained by retinoscopy in routine care, eyes near the 0.25 D/year threshold may have been misclassified. Serial axial length data were not available, limiting structural confirmation of treatment response. Adherence and side effects were based on family report and may be subject to recall bias. Predicted ethnicity was derived from BISG rather than self-identified race/ethnicity. Because BISG provides only probabilistic estimates, individual-level misclassification is possible; however, ethnicity was used only as an adjustment covariate, so any resulting misclassification would be expected to attenuate ethnicity-related inference rather than materially alter the primary treatment-signal results. Treatment selection, escalation, and discontinuation were not randomized; residual confounding by indication is unavoidable despite adjustment and event-time sensitivity analyses. Because this referral-practice cohort includes children with secondary or comorbidity-associated myopia not typically represented in randomized myopia-control trials, absolute progression rates should not be directly compared with those reported in trial-eligible community myopia populations. Finally, some subgroup analyses (particularly PD-Glasses initiation and certain comorbidities) were limited by small paired samples and should be considered exploratory, and generalizability may be most applicable to similar US practice settings.

Despite these limitations, this cohort provides a detailed view of adaptive, real-world myopia management in a US practice over relatively long follow-up. The integrated pattern of findings supports a cohesive clinical message: 12-month refractive response is a practical prognostic marker for whole-course control and a management signal to guide escalation, not an immutable success/failure label. In routine care, response-guided escalation is associated with substantial slowing among initial failures and attenuation of post-12-month differences, while escalation in already controlled eyes shows little incremental benefit. These results support a dynamic, individualized approach in which first-year progression informs counseling, follow-up intensity, and timely escalation decisions to optimize long-term myopia control while minimizing unnecessary treatment burden.

## Declaration of Generative AI and AI-Assisted Technologies in the Writing Process

During preparation of this work, the authors used ChatGPT-5.2 in order to improve the language and readability of the manuscript. After using this service, the authors reviewed and edited the content as needed and take full responsibility for the content of the publication.
